# Gastrointestinal manifestation as clinical predictor of severe COVID‐19: A retrospective experience and literature review of COVID‐19 in Association of Southeast Asian Nations (ASEAN)

**DOI:** 10.1002/jgh3.12394

**Published:** 2020-09-12

**Authors:** Natsuda Aumpan, Pongjarat Nunanan, Ratha‐korn Vilaichone

**Affiliations:** ^1^ Gastroenterology Unit, Department of Medicine, Faculty of Medicine Thammasat University Hospital Khlong Nueng Thailand; ^2^ Department of Medicine Chulabhorn International College of Medicine (CICM), Thammasat University Khlong Nueng Thailand; ^3^ Digestive Diseases Research Center (DRC) Thammasat University Hospital Khlong Nueng Thailand

**Keywords:** Association of Southeast Asian Nations (ASEAN), COVID‐19, gastrointestinal manifestations

## Abstract

**Background and Aim:**

Coronavirus disease 19 (COVID‐19) has caused over 200 000 deaths worldwide. Thailand announced the first confirmed case outside mainland China in January 2020. The disease also spread widely across Association of Southeast Asian Nations (ASEAN). Gastrointestinal manifestations could be presenting symptoms of COVID‐19. This study aimed to determine the prevalence of gastrointestinal manifestations of COVID‐19 patients in Thailand and review important aspects of this disease in ASEAN.

**Methods:**

Thai patients diagnosed with COVID‐19 at Thammasat University Hospital, Thailand, were evaluated between 1 January 2020 and 30 April 2020. Patients' data, clinical presentation, exposure risk, past medical history, laboratory results, and treatment outcomes were extensively reviewed.

**Results:**

A total of 352 COVID‐19 tests were performed, and 40 patients with positive tests were studied. The mean age was 30.5 years, and 55% were female. Most (82.5%) had no underlying diseases. Comorbidities were associated with severe COVID‐19 (odds ratio [OR] 29.93; 95% confidence interval [CI] 2.31–388.40, *P* = 0.009). Gastrointestinal symptoms were present in 12 patients (30%). The most common presenting symptoms were anorexia (17.5%) and diarrhea (15%). Gastrointestinal symptoms developed before (9.1%), concurrent with (63.6%), and after (27.3%) respiratory symptoms. Diarrhea was significantly associated with severe COVID‐19 (OR 38.52; 95% CI 3.11–476.70, *P* = 0.004). Twenty‐four patients (60%) received antiviral drugs; 40% had only supportive care. Only one patient required intensive care. No patient died.

**Conclusions:**

Gastrointestinal manifestations in COVID‐19 patients are common symptoms and can occur anytime during the disease course. Patients presenting with only gastrointestinal symptoms should raise clinical suspicion for COVID‐19 in areas with high disease incidence. Clinically severe COVID‐19 was associated with comorbidities and diarrhea.

## Graphical abstract

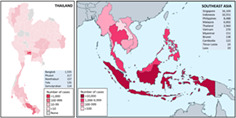

## Introduction

Severe acute respiratory syndrome coronavirus 2 (SARS‐CoV‐2) is an enveloped, single‐stranded, positive‐sense RNA virus with high transmissibility.^1^ It has caused a pandemic, named coronavirus disease 19 (COVID‐19), that has affected more than 3 million people and caused over 200 000 deaths worldwide. The wide spectrum of the disease ranges from asymptomatic infection to severe pneumonia requiring mechanical ventilation.^2^ The majority of patients have respiratory manifestations, and many report gastrointestinal symptoms.^3^, ^4^, ^5^ Anorexia, diarrhea, abdominal pain, nausea, and vomiting are reported in 3–79% of patients.^6^ There are abundant viral receptor expressions on gastrointestinal epithelial cells, and recent studies have suggested possible fecal–oral transmission.^7^, ^8^, ^9^ It has been suggested that preventive measures against fecal contamination should be implemented owing to prolonged duration of viral shedding in stool up to 16 days from symptom onset.^10^

The Association of Southeast Asian Nations (ASEAN) is composed of 11 countries with a total population of more than 655 million people, ranking third among all subregions in the world. The coronavirus outbreak was recognized in Wuhan, China, in December 2019. Thailand announced the first confirmed case outside mainland China during the Chinese New Year on 13 January 2020, and the disease continued to spread widely across ASEAN.^11^ Until 30 April 2020, Thailand recorded 66 371 patients undergoing COVID‐19 tests, and 2954 tested positive. According to data from the Thai Ministry of Public Health, Bangkok, the capital city of Thailand, had the highest number of COVID‐19 cases (51.9%) in the country, followed by Phuket (7.3%), the popular island resort in the south.

COVID‐19 has continued to be responsible for high health‐care resource utilization and economic burden throughout ASEAN. This study aimed to determine the prevalence of gastrointestinal manifestations of COVID‐19 patients in Thailand and review important aspects of this disease in ASEAN.

## Methods

### *Study design*

This retrospective study was conducted at Thammasat University Hospital between 1 January 2020 and 30 April 2020. The inclusion criteria were patients aged more than 15 years old who were diagnosed with COVID‐19. All patients in this study were of Thai ethnicity. Demographic data, clinical presentation, exposure risk, past medical history, laboratory results including a complete blood count and a comprehensive metabolic panel, and treatment outcomes were extracted from a medical database and extensively reviewed. The clinical data and blood investigation were recorded within 24 h of hospital presentation. Data were analyzed and interpreted by the authors.

### *Definition*

*The diagnosis of COVID‐19* was defined as confirmed detection of SARS‐CoV‐2 in either upper or lower respiratory tract samples using real‐time reverse‐transcription polymerase chain reaction (RT‐PCR). Upper respiratory tract samples could be obtained through nasopharyngeal wash/aspirate, nasal wash/aspirate, or nasal swab. The lower respiratory tract specimens were obtained using tracheal aspirate or bronchoalveolar lavage.^12^

*Comorbidity* was defined as the presence of one or more underlying medical conditions (e.g. diabetes mellitus, hypertension, dyslipidemia, etc.) in addition to a current diagnosis of COVID‐19.

*Occupational risk for COVID‐19* was defined as including health‐care workers, retail store workers, tourism staff, bus or taxi drivers, multinational business staff, transport and security workers, and construction workers.^13^

*Incubation period* was defined as the time between exposure to SARS‐CoV‐2 and symptom onset.^14^

*COVID‐19 with mild symptoms* was defined as having a normal chest radiograph according to the Thai practice guideline for diagnosis, treatment, and prevention of nosocomial transmission of COVID‐19 (revision date 1 May 2020).^15^

*COVID‐19 with severe symptoms* was defined as having signs and symptoms of pneumonia or an abnormal chest radiograph.^15^ The abnormality could be either consolidation or ground‐glass opacity. The extent of abnormality could be at the peripheral area, lower lung zone, or bilateral involvement.^16^

*Gastrointestinal manifestations* of COVID‐19 included anorexia, nausea and vomiting, watery diarrhea, bloody diarrhea, or abdominal pain.

*Diarrhea* was defined as an abrupt onset of the passage of ≥3 loose stools per day.^17^

### *Statistical analysis*

All data were analyzed using SPSS version 22 (SPSS Inc., Chicago, IL, USA). The demographic data were analyzed by Fisher's exact test or Chi‐square test, where appropriate. Laboratory results were compared between patients with mild symptoms and those with severe symptoms using Student's t‐test. The univariate and multivariate analyses were used for determining the association between factors and severity of symptoms. A *P* value of <0.05 was indicated statistical significance. Ethical approval for this study was obtained from the Human Research Ethics Committee of Thammasat University, Thailand. The research was conducted according to the good clinical practice guideline, as well as the Declaration of Helsinki.

## Results

### *Demographic data*

Forty Thai patients were studied, including 18 men and 22 women, with a mean age of 30.5 ± 9.2 (range 20–55) years. The majority (82.5%) had no underlying medical conditions. The presence of comorbidities was associated with more severe symptoms of COVID‐19 (odds ratio [OR] 29.93; 95% confidence interval [CI] 2.31–388.40, *P* = 0.009). The diagnosis of COVID‐19 was confirmed by positive RT‐PCR assays of respiratory specimens in all patients. In Thammasat University Hospital, a total of 352 tests were performed, and 40 patients tested positive. All 40 patients were admitted to Thammasat University Hospital, a major referral center located north of Bangkok. Most patients in this study had close contact with a confirmed case (40%), followed by unknown exposure (30%). The mean incubation period of patients with mild symptoms was not different from those with severe disease (6.4 ± 4.4 *vs* 4.5 ± 2.2 days, *P* = 0.327). The mean time from symptom onset to hospital admission was also not different between groups of mild and severe symptoms (9.6 ± 5.4 *vs* 8.1 ± 5.1 days, *P* = 0.448).

**Figure 1:**
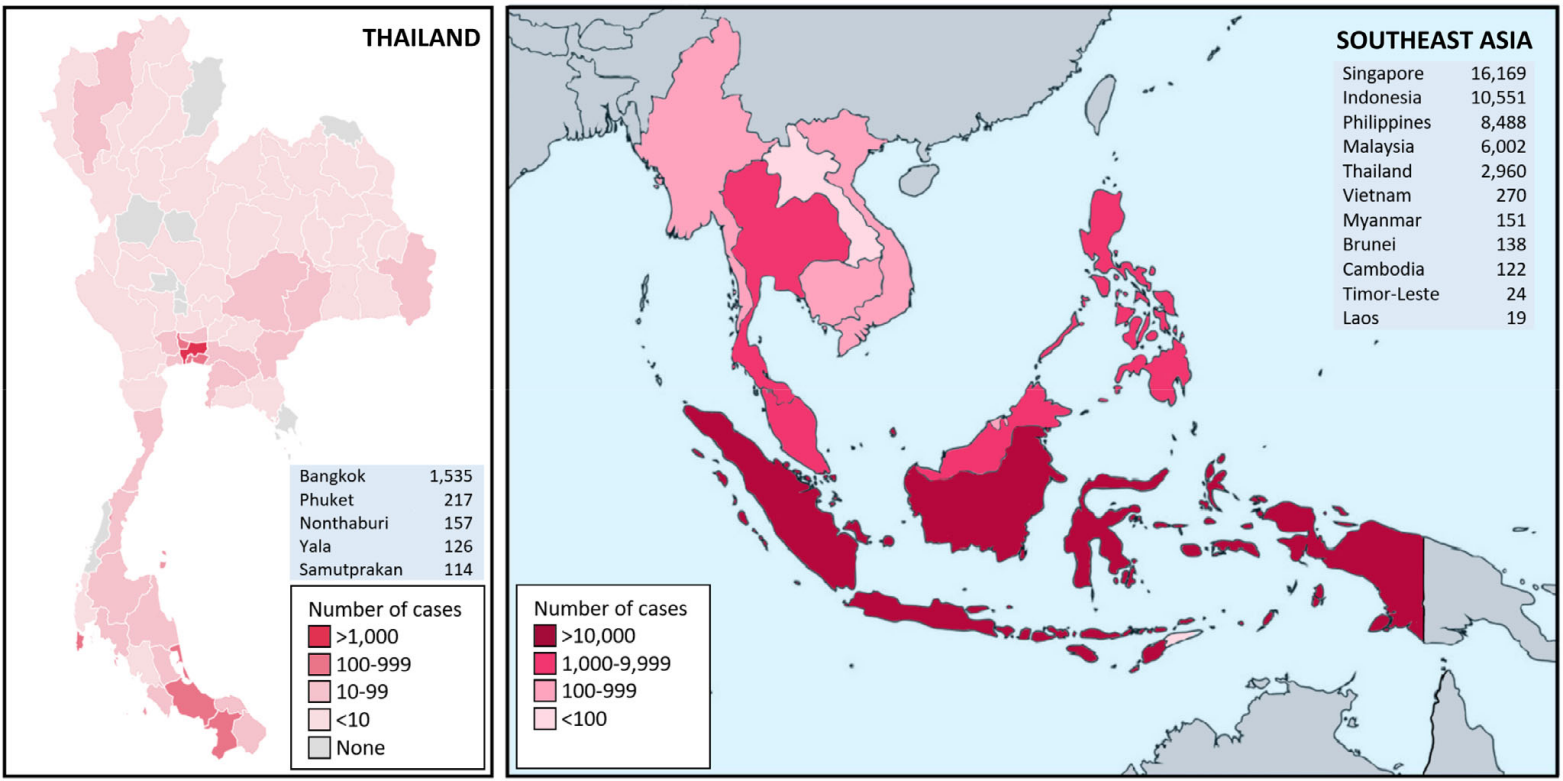
The geographic distribution of COVID‐19 patients in Thailand (left) and ASEAN (right). The official data of all confirmed cases in Thailand were retrieved from the Department of Disease Control, Ministry of Public Health, Thailand, as of 1 May 2020. The official data of all confirmed cases in ASEAN were obtained from World Health Organization as of 1 May 2020.

**Figure 2:**
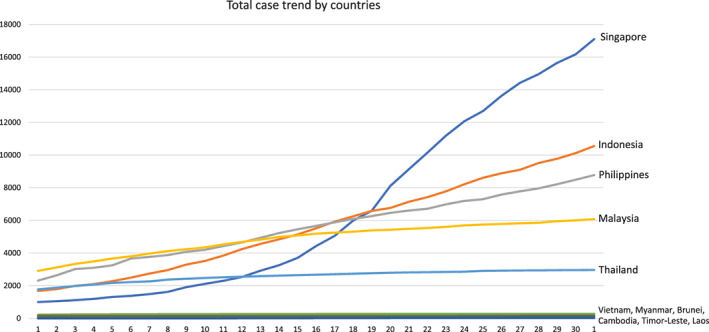
Total case trend in ASEAN. The official data of all confirmed cases in ASEAN were obtained from World Health Organization between 1 April 2020 and 1 May 2020.

The geographic distribution of COVID‐19 patients in Thailand and ASEAN is shown in Figure 1. The trend of total cases in ASEAN was demonstrated in Figure 2. Demographic data of COVID‐19 patients classified by severity of symptoms are given in Table 1. Multivariate analysis of associated gastrointestinal risk factors with severity of symptoms is elucidated in Table 2.

**Table 1:** Demographic data of COVID‐19 patients classified by severity of symptoms

Characteristics	Mild symptoms (*n* = 30)	Severe symptoms (*n* = 10)	Odds ratio (95% CI)	*P* value
Mean age (years ± SD)	29.7 ± 8.7	33.0 ± 10.8		0.335
Female, *n* (%)	16 (53.3)	6 (60.0)	1.31 (0.31–5.62)	0.714
Comorbidity, *n* (%)	3 (10.0)	4 (40.0)	6.00 (1.05–34.14)	0.043
Exposure, *n* (%)			—	—
Contact of confirmed case	12 (40.0)	4 (40.0)		
International travel	1 (3.3)	1 (10.0)		
Domestic travel	2 (6.7)	1 (10.0)		
Occupational risk	6 (20.0)	1 (10.0)		
Unknown	9 (30.0)	3 (30.0)		
Mean incubation period (days ± SD)	6.4 ± 4.4	4.5 ± 2.2	—	0.327
Mean onset of symptoms to admission (days ± SD)	9.6 ± 5.4	8.1 ± 5.1	—	0.448
Clinical presentation				
GI symptoms				
Anorexia	5 (16.7)	2 (20.0)	1.25 (0.20–7.74)	0.810
Nausea and vomiting	0 (0)	2 (20.0)		0.058
Diarrhea	2 (6.7)	4 (40.0)	9.33 (1.38–63.20)	0.022
Abdominal pain	1 (3.3)	1 (10.0)	3.22 (0.18–56.88)	0.424
Non‐GI symptoms				
Fever (≥37.5°C)	4 (13.3)	4 (40.0)	4.33 (0.84–22.47)	0.081
Cough	20 (66.7)	7 (70.0)	1.17 (0.25–5.50)	0.846
Sore throat	16 (53.3)	3 (30.0)	0.38 (0.08–1.73)	0.209
Nasal congestion	12 (40.0)	3 (30.0)	0.64 (0.14–2.99)	0.573
Myalgia	10 (33.3)	2 (20.0)	0.50 (0.09–2.81)	0.431
Headache	4 (13.3)	0 (0)	—	0.556
Fatigue	0 (0)	6 (60.0)		<0.001
Dyspnea	1 (3.3)	6 (60.0)	43.50 (4.10–461.19)	0.002
Laboratory findings				
White cell count (×10^9^/L)	6.0 ± 1.7	8.2 ± 2.6		0.013
Neutrophil count (×10^9^/L)	3.0 ± 1.3	5.3 ± 2.6		0.007
Lymphocyte count (×10^9^/L)	2.3 ± 0.8	2.0 ± 0.5		0.292

CI, confidence interval; GI, gastrointestinal; SD, standard deviation.

Significant factors for shading are comorbidity, diarrhea, fatigue, dyspnea, white cell count, and neutrophil count.

**Table 2:** Multivariate analysis of associated gastrointestinal risk factors with severity of symptoms

Risk factors	Odds ratio (95% CI)	*P* value
Female	1.75 (0.27–11.33)	0.560
Comorbidities	29.93 (2.31–388.40)	0.009
Diarrhea	38.52 (3.11–476.70)	0.004

CI, confidence interval.

Significant factors for shading are comorbidities and diarrhea.

### *Gastrointestinal manifestations*

There were 12 patients (30%) with gastrointestinal symptoms. The most common presenting gastrointestinal symptoms were anorexia (17.5%), followed by diarrhea (15%). Nausea and vomiting and abdominal pain were observed equally at 5%. None had bloody diarrhea. Gastrointestinal symptoms could develop before (9.1%), concurrent with (63.6%), or after (27.3%) respiratory symptoms. Patients most frequently had only one gastrointestinal symptom (75%). Of nine patients with a single gastrointestinal symptom, five patients (55.6%) had anorexia, and four (44.4%) had diarrhea. There was one patient with all five gastrointestinal manifestations. That patient was a 51‐year‐old woman without comorbidity. She had close contact with her husband who died of complications from COVID‐19 pneumonia. Two weeks before admission, she had intermittent low‐grade fever with myalgia and reported loss of appetite. She developed shortness of breath, productive cough, watery diarrhea, and epigastric pain 2 days before admission. She was subsequently diagnosed with pneumonia. Her chest radiograph demonstrated bilateral reticular infiltration with predominant right lower lobe consolidation. She was started on darunavir, ritonavir, favipiravir, hydroxychloroquine, and azithromycin on the first day of admission. During hospitalization, she had nausea and vomiting. All symptoms subsided after 10 days of admission. This patient recovered and was discharged from the hospital 23 days after admission. Another patient had only gastrointestinal symptoms without respiratory abnormality. This patient was a 31‐year‐old man with COVID‐19 gastroenteritis and was admitted with generalized abdominal discomfort, bloating, and watery diarrhea. He denied having fever or respiratory symptoms. He received antiviral treatment for 10 days, and his symptoms improved. This patient was discharged after 11 days of admission. In multivariate analysis, diarrhea was significantly associated with severe symptoms of COVID‐19 (OR 38.52; 95% CI 3.11–476.70, *P* = 0.004). Clinical symptoms of COVID‐19 patients in ASEAN are given in Table 3.

**Table 3:** Clinical symptoms of COVID‐19 in ASEAN (*n* = 183)

			GI symptoms				Non‐GI symptoms			
Country	Study	Total patients	Diarrhea (%)	Nausea (%)	Vomiting (%)	Abdominal pain (%)	Fever (%)	Cough (%)	Sore throat (%)	Dyspnea (%)
Thailand	This study	40	15	5	5	5	20	67.5	47.5	17.5
Thailand	Pongpirul *et al*.^18^	11	18	NA	27	NA	91	91	82	NA
Thailand	DDC, MOPH, Thailand^19^	30	3	NA	7	NA	83	70	40	13
Singapore	Pung *et al*.^20^	17	24	6	6	NA	88	82	47	35
Singapore	Young *et al*.^21^	18	17	NA	NA	NA	72	83	61	11
Singapore	Sun *et al*.^22^	54	37				NA	66.7	33.3	13
Indonesia	Tenda *et al*.^23^	3	33	0	0	NA	100	33	NA	33
Indonesia	Azwar *et al*.^24^	1	0	100	100	100	100	100	NA	100
Malaysia	See *et al*.^25^	4	25	NA	NA	NA	50	50	NA	NA
Vietnam	Than *et al*.^26^	5	NA	NA	NA	NA	80	100	40	0

DDC, MOPH = Department of Disease Control, Ministry of Public Health, Thailand; NA, not applicable.

### *Nongastrointestinal manifestations*

The most common presenting symptoms were cough (67.5%), sore throat (47.5%), and nasal congestion (37.5%). Fever defined as body temperature ≥37.5°C was present in 20% of patients. Headache was present only in patients with mild symptoms, whereas fatigue was found only in the group with severe symptoms. In this study, patients were mostly diagnosed with nasopharyngitis (37.5%), followed by pneumonia (25%), pharyngitis (22.5%), and bronchitis (12.5%).

### *Radiologic and laboratory results*

Patients (10) with pneumonia had abnormal findings on chest radiographs. Six had focal ground‐glass opacity, and the others had diffuse ground‐glass opacities. Patients with severe symptoms were inclined to have more white blood cells (8.2 ± 2.6 × 10^9^/L *vs* 6.0 ± 1.7 × 10^9^/L, *P* = 0.013) and neutrophil counts (5.3 ± 2.6 × 10^9^/L *vs* 3.0 ± 1.3 × 10^9^/L, *P* = 0.007) than the mild symptom group. Lymphopenia, defined as lymphocyte count of less than 1.5 × 10^9^/L, was present in three patients with mild symptoms and one patient with severe symptoms. There was no difference in renal or liver function test between patients with mild and severe symptoms.

### *Treatment outcomes and complications*

Twenty‐four patients (60%) received antiviral drugs, including either darunavir/ritonavir or lopinavir/ritonavir, favipiravir, hydroxychloroquine, and azithromycin; 40% had only supportive care. Thirty‐nine patients demonstrated improvement of their symptoms without any complications and were discharged home after a mean of 16.5 ± 7.8 days. One patient had a complication from COVID‐19. A 37‐year‐old woman with hypertension had acute dyspnea, nasal congestion, and productive cough for 1 day before admission. She denied having any fever, myalgia, or gastrointestinal symptom. She was diagnosed with pneumonia and required ventilator support for respiratory failure. Medications including darunavir/ritonavir, favipiravir, hydroxychloroquine, and azithromycin were immediately started. On the same day, she developed myocarditis and cardiogenic shock. The echocardiogram showed global hypokinesia with left ventricular ejection fraction of 20%. One gram of methylprednisolone daily for 3 consecutive days was started, along with inotropic drugs and diuretics. Her symptoms gradually improved. She was extubated 6 days after admission and was later discharged home. No mortality was observed in this study.

## Discussion

The COVID‐19 pandemic has had an enormous impact on global community since January 2020. The ASEAN was one of the first sites where the virus began spreading, and the number of the infected patients in this region has increased rapidly.^11^ In Thailand, more than half of confirmed cases have been in Bangkok or were predominantly clustered in southern provinces (Phuket, Yala, and Songkhla) and Bangkok's surrounding areas (Nonthaburi and Samutprakan). While other studies demonstrated a higher proportion of men, our study had slightly more women than men.^2^, ^27^ This study also confirmed that the presence of comorbidities was significantly associated with more severe symptoms of COVID‐19.^3^

The gastrointestinal tract is affected by SARS‐CoV‐2 infection.^7^, ^8^ Angiotensin‐converting enzyme 2 (ACE2) receptor and transmembrane serine protease 2 (TMPRSS2) are required for viral entry into host cells. Apart from alveolar cells in lung tissue, ACE2 and TMPRSS2 are highly coexpressed in glandular epithelial cells in the esophagus, ileum, and colon.^8^ The mechanism through which SARS‐CoV‐2 causes gastrointestinal symptoms is not clearly understood but is suspected to be involved with ACE2 function. ACE2 naturally regulates homeostasis of intestinal amino acids and gut microbiomes. Altered ACE2 function can result in amino acid malnutrition and diarrhea as demonstrated in ACE2 knockout mice.^28^ The prevalence of gastrointestinal manifestations in COVID‐19 patients in this study was 30%, which was relatively higher than other studies in China.^2^, ^3^, ^27^ However, prior studies from Singapore showed comparable prevalence of overall gastrointestinal symptoms (37%) and specific symptom such as diarrhea (17–24%).^20^, ^21^, ^22^ Therefore, COVID‐19 patients in ASEAN appear to be more inclined to have gastrointestinal symptoms. The proportion of patients with diarrhea (15%) in this study was almost the same as an earlier study in Thailand (18%).^18^ Moreover, our study demonstrated that diarrhea was significantly associated with severe symptoms. The previous study showed that abdominal pain and anorexia were associated with more severe symptoms, while diarrhea was not different between groups.^3^ Diarrhea was also described in COVID‐19 pediatric patients with mild symptoms in Malaysia.^25^ One patient in our study exhibited only gastrointestinal symptoms, and another patient had diarrhea before the onset of respiratory symptoms. Neither patient had fever. The time from onset of gastrointestinal symptoms to hospital admission was 15 and 12 days, respectively. In addition, a case report described an unusual presentation of COVID‐19 in a young Indonesian woman with predominant epigastric and chest pain, which was initially treated as gastroesophageal reflux disease.^24^ One day later, she developed dyspnea and was diagnosed with COVID‐19 pneumonia. More attention should be focused on COVID‐19 patients presenting with only digestive symptoms because of the possible higher risk of transmission during an undiagnosed period.^29^ The most common nongastrointestinal symptom was cough (67.5%), which was similar to previous studies.^22^, ^27^ Sore throat was the second most common symptoms in ASEAN (33–82%), whereas China had a lower incidence of this symptom (13.9–17.4%).^3^, ^18^, ^27^

In conclusion, gastrointestinal manifestations in COVID‐19 patients are common presenting symptoms and can occur anytime during the disease course, particularly in ASEAN. Patients presenting with only gastrointestinal symptoms should raise clinical suspicion for COVID‐19 in areas with high disease incidence. Comorbidities and diarrhea might be good predictors for the development of severe COVID‐19.
